# Predictors of recurrence of chronic subdural haematoma in a cohort study of patients presenting in a sub-Saharan African teaching hospital

**DOI:** 10.1186/s12883-022-02857-1

**Published:** 2022-09-14

**Authors:** H. M. Abdullah, T. Dakurah, H. Akoto, B. Abaidoo, J. C. B. Dakubo, A. E. Yawson, G. Wepeba, P. Bankah, J. Boatey, M. Ametefe, F. Nketiah-Boakye, A. Fuseini, M. Banson, T. Ndanu, A. Mubarak, M. Iddrissu

**Affiliations:** 1 Department of Surgery, Korle Bu Teaching Hospital, Korle Bu, Accra, Ghana; 2 University of Ghana Medical School, College of Health Sciences, University of Ghana, Accra, Ghana; 3 Opthalmology Unit, Department of Surgery, University of Ghana Medical School, University of Ghana, Accra, Ghana; 4 Komfo Anokye Teaching Hospital, Kumasi, Ghana

**Keywords:** Chronic, Subdural, Haematoma, Recurrence, Predictors, Association

## Abstract

**Background:**

Chronic subdural haematoma (CSDH) is a common neurological condition affecting the elderly with decreased quality of life. Recurrence leads to increase in number of hospital admissions and surgical interventions. Several factors contribute to recurrence of chronic subdural haematoma, and determination of these factors will help institute measures to reduce recurrence of CSDH, cost of care and improved quality of life. The aim of this study was to determine the predictors of recurrence of chronic subdural haematoma in a cohort of patients presenting in a Sub-Saharan African Teaching Hospital.

**Methods:**

A prospective hospital-based cohort study of 62 participants who presented with CSDH and underwent burr-hole and drainage at the Neuroscience unit of the Korle-bu Teaching Hospital. The primary outcome of this study was the recurrence of CSDH within 3 months after the surgery. Data was entered into Microsoft Excel 2016 and exported to International Business Machine (IBM) Statistical Package for the Social Sciences (SPSS) version 21.0 for analysis. Predictors of recurrence of CSDH were determined using logistic regression with odds ratio calculated at the 95% confidence level and a *p*-value less than 0.05 accepted as statistically significant.

**Results:**

There was a male preponderance of 45 (72.6%), over females of 17 (27.4%). The mean age was 63.1 ± 13.6 years. The recurrence rate of CSDH was 21.0% whilst the mortality rate was 4.8%. Facial palsy and dysphasia were associated with the recurrence of CSDH (*p* = 0.045, 0.029). Hypertension and bilaterality were associated with recurrence of CSDH from a univariate analysis (*p* = 0.039, OR = 4.865, CI = 0.975–24.285; *p* = 0.005, OR = 5.979, CI = 1.585–22.557). In a multivariate logistic regression analysis, bilaterality was the only independent predictor of recurrence of CSDH (*p* = 0.030, AOR = 5.47, CI = 1.18–25.34).

**Conclusions:**

Both hypertension and bilaterality showed statistically significant association with recurrence of CSDH. However, only bilaterality proved to be an independent predictor of recurrence of CSDH in patient who underwent burr-hole and drainage.

**Supplementary Information:**

The online version contains supplementary material available at 10.1186/s12883-022-02857-1.

## Background

Chronic subdural haematoma (CSDH) is one of the most common neurological conditions which affect the elderly [1]. The incidence of chronic subdural haematoma is 58/100,000 in those aged above 65 years [1]. CSDH is a major cause of neurological morbidity and mortality [2]. Even though some patients improve rapidly with surgical intervention, the neurological status may worsen in about 4% of them [3]. CSDH has a mortality rate of up to 11% and this may be associated with the extent of the surgical intervention [2, 3]. The morbidity and mortality rates depend on the patient’s age, co-morbidities and the surgical techniques used in evacuating the haematoma [3].

In a systematic review and meta-analysis of radiological prognostic factors of chronic subdural hematoma, recurrence was reported to be 14.4% [4]. Many factors are cited as determinants of recurrence following evacuation of haematoma [5, 6]. High recurrence rates are associated with increase in reoperations for CSDH with decreased quality of life [7].

Both anticoagulants and antiplatelets use are associated with an increased risk in the development of CSDH [8, 9]. In spite of the aforementioned, their role in the development of recurrence of CSDH has not been settled. Three separate studies concluded differently on previous use of anticoagulant or antiplatelets and their association with the development of recurrence of chronic subdural haematoma [10, 11].

The contributions of co-morbidities (hypertension and diabetes) to recurrence of CSDH is similar to that of anticoagulant and antiplatelet use. Patients with hypertension and diabetes are frequently on these medications as treatment or prophylaxis for cardiovascular and cerebrovascular events. Findings in previous retrospective studies have been equivocal [12, 13]. The prevalence of hypertension in Ghana has been estimated to be between 20 to 25% [14]. The 2017 International Federation of diabetes’ report indicates that the prevalence of diabetes mellitus amongst Ghanaians is 3.6% and this has been projected to quadruple by the year 2030 [15]. With the current prevalence of hypertension and diabetes, and the expected increase in the population of Ghanaians above 65 years, the incidence of CSDH may increase [2].

At the Neuroscience Unit of the Korle-Bu Teaching Hospital, chronic subdural haematoma is currently the most common cranial emergency which requires surgical intervention. Averages of 4 cases of chronic subdural hematomas are seen at the center every month. The improvement in availability of Computerised Tomography (CT) scanners and Magnetic Resonance imaging (MRI) equipment across the country is expected to improve the diagnosis of this disease. Improved diagnosis and increase in the population of the elderly in the country will likely lead to a rise in the incidence of chronic subdural haematoma and hence the number of surgeries performed. Although risk factors of recurrence of CSDH remain a controversy, the determination of these factors will help institute measures that will reduce recurrence, the burden of the disease, cost of care and improvement in the quality of life in those patients. This study therefore aimed at determining the predictors of recurrence of chronic subdural haematoma in a Sub-Saharan African Teaching Hospital.

## Methods

### Study design

This was a prospective hospital-based cohort study of patients with chronic subdural haematoma who had burr-hole craniostomy and drainage at the Neuroscience Unit of the Korle-Bu Teaching Hospital.

### Study population

The study was conducted from April 2018 to May 2019. The study population was all patients with CSDH who reported to or were referred to the Neuroscience unit of the Korle-Bu Teaching Hospital and required surgical intervention between April 2018 and May 2019.

### Inclusion and exclusion criteria

Patients with CT scan or MRI confirming chronic subdural haematoma referred to the Neuroscience Unit of Korle-Bu Teaching Hospital who had burr-hole and evacuation were included in the study. The following patients were excluded from the study:

1. Patients who undergone surgical procedures other than burr-hole craniostomy. These included craniotomy, twist drill and use of the subdural evacuation port (SEP) device. Though the center has the capacity to perform these procedures, they are rarely done. Many patients cannot afford the SEP device.
2. Patients with bilateral chronic subdural haematoma who had drainage of only one haematoma site
3. Patients with ventriculoperitoneal (VP) shunt in-situ.
4. Patients with a history of stroke.
5. Patients with hydrocephalus.
6. Patient with multi-layered type of CSDH
7. Patients who died in the immediate post-operative period, i.e. less than 24 hours after surgery.

### Sample size calculation

Using 80% power and 10% attrition, a sample size of 62 was obtained considering the calculated mean of 3.6% for the West African sub-region and an expected higher recurrence rate for KBTH, 20 years after the study by Dakurah et al. [16, 17].

### Procedure

Patients referred to the Neuroscience Unit of the Korle-Bu Teaching Hospital who met the inclusion criteria during the study period were recruited consecutively until sample size was obtained. After obtaining an informed written consent, the researchers administered the admission proforma and directly filled on the demographic characteristics of patients, history of hypertension and diabetes, use of anticoagulants, anti-platelets and abuse of alcohol. Data on the size, laterality and degree of midline shift on radio-imaging was collected. All patients had their clotting profile and platelets counts evaluated before surgery. Patients with Glasgow coma outcome scores (GCS) of 15 on anticoagulants with International Normalised Ratio (INR) above 2 received vitamin K for a period of 5 days and had their INR repeated before surgery. For deteriorating patients who needed emergency surgery, Fresh frozen plasma was given prior to the emergency procedure. Patients on antiplatelets such as aspirin were observed for a period of 7 days before surgery. In this case, the aspirin was stopped. In the cases of emergency, platelet concentrate was transfused before surgery.

In the operating theater, patients were positioned supine on a headrest. The incision site was infiltrated with 10mls of 1% xylocaine plus 1:200,000 adrenaline. Under cardiopulmonary monitoring, a 2.0 cm burr-hole was drilled over the maximum width of the haematoma. The dura mater was coagulated. It was then opened with a cruciate incision and the resulting cusps coagulated. A maximum of size 12 French nelaton drainage tube was passed into the subdural space and its distal end connected to a bulb syringe. The subdural collection was then washed out with normal saline till the effluent was clear. Bilateral subdural haematoma was treated the same with each side evacuated, starting with the larger one first. Each drain was connected to a soft collection bag that was hanged on the side of the bed and removed at 72 hours regardless of the amount of fluid drained. However, if the effluent was Cerebrospinal fluid (CFS), the drainage tube was removed regardless of the time and amount.

The primary outcome of this study was the recurrence of CSDH within 3 months after the surgery. Each patient had repeat CT scan of the brain at 1 month and 3 months after surgical evacuation of CSDH.

Recurrence was defined as a case of reoperation in a symptomatic patient due to an increase in hematoma volume or thickness and/or presence of hyperdensity in the ipsilateral subdural space seen on CT scan within 3 months’ post-operative period. Patients who reported with signs and symptoms suggestive of raised intracranial pressure had an urgent CT scan of the brain. Patients who had a radiologically increasing hematoma with symptoms of raised intracranial pressure or neurological deficits due to the haematoma underwent re-operation.

The study evaluated the demographic characteristics of patients, the clinical presentation including the functional status of the patients using the modified Rankin score which were recorded on a predesigned template. At admission, the presence of medical condition such as hypertension and diabetes, information on location, size of the haematoma and midline shift, clotting profiles, platelets counts, were captured on the template. The modified Rankin score (Additional file 1) and Glasgow outcome score (Additional file 1) were used to evaluate the functional outcome of patient 3 months after surgery. Tools are attached as an Additional file 1.

#### Statistical analysis

Data was entered in Microsoft Excel 2016 and imported into IBM SPSS version 21.0 for analysis. Outcome measures of interest such as demographic characteristics, clinical history etc. were analyzed using descriptive statistics (e.g. proportions, frequencies, ratios). Continuous measures such as INR, platelets count, size of haematoma, and degree of midline shift were summarized as mean and standard deviation. Categorical variables were analysed using the Chi-square test or the Fishers Exact test. Functional outcomes were evaluated using Glasgow coma outcome scores and modified Rankin scores. A Glasgow outcome score of 4 and 5 were designated as a good outcome. Poor outcomes were scored less than 4. A Modified Rankin score of 0 to 2 was designated as a good outcome while scores above 2 was poor outcome. Predictors of recurrence of CSDH such as the use of antiplatlets, anticoagulants and alcohol, hypertension and diabetes, were determined using logistic regression with odds ratio calculated at the 95% confidence level and a *p*-value less than 0.05 accepted as statistically significant.

## Results

A total of 62 participants were enrolled in this study. There was a male preponderance of 45 (72.6%), over females of 17 (27.4%). The youngest participant was 33 years and the oldest 88 years old. The mean age of the participants was 63.1 ± 13.6 years. Majority (38, 61.3%) of the participants were 60 years and beyond whilst 24 (38.7%) were below 60 years, Table 1.

**Table 1:** Association between recurrence of CSDH and demographics

Characteristics	Recurrence		Total N (%)	X^2/^Fisher’s exact- value	*P*-value
	Yes n (%)	No n (%)			
Age				7.421^a^	0.208
< 40	–	4 (6.5)	4 (6.5)		
41–50	–	7 (11.3)	7 (11.3)		
51–60	2 (3.2)	12 (19.4)	14 (22.6)		
61–70	3 (4.8)	13 (21.0)	15 (25.8)		
71–80	6 (9.7)	9(14.5)	15 (24.2)		
> =81	2 (3.2)	4 (6.5)	6 (9.7)		
Total	13 (21.0)	49 (79.0)	62 (100.0)		
Sex				0.156^a^	0.495
Male	10 (16.1)	35 (56.5)	49 (79.0)		
Female	3 (4.8)	14 (22.6)	17 (27.4)		

^a^ Fisher’s exact test, X^2^ chi-square value

Fifty (80.6%) participants presented with headache, forty-seven (75.8%) had hemiparesis and forty-one (66.1%) had facial palsy. Thirty-six (58.1%) had dysphasia and only 8 (12.9%) presented with seizure, Table 2.

**Table 2:** Association between clinical presentation and recurrence of CSDH

Clinical presentation	Recurrence		Test statistics	*P*-value
	No recurrence N(%)	Recurrence N(%)		
Headache:			0.146^a^	0.27
No	9(14.5)	3(4.8)		
Yes	40(64.5)	10(16.1)		
Seizure:			2.282^a^	0.12
No	45(72.6)	10(16.1)		
Yes	4(6.5)	3(4.8)		
Hemiparesis:			0.487^a^	0.25
No	12(19.4)	2(3.2)		
Yes	37(59.7)	11(17.7)		
Facial palsy:			5.033^a^	0.045
No	20(32.3)	1(1.6)		
Yes	29(46.8)	12(19.4)		
Dysphasia:			4.762^a^	0.029
No	24(38.7)	2(3.2)		
Yes	25(40.3)	11(17.7)		

^a ^Fisher’s exact test

Majority (56.5%) of the participants had a history of trauma. For those with history of trauma, 10 (16.1%) had road traffic accident, 20 (32.3%) had a fall, 4 (6.5%) accidentally hit their heads against an object, and 1 (1.6%) participant was assaulted. One (1.6%) participant who was a known epileptic had an episode of seizure 3 weeks earlier.

Out of the 62 participants, 37 (59.7%) were hypertensive, 8 (12.9%) had diabetes mellitus, and 6 (9.7%) used alcohol. Eleven (9.7%) were on antiplatelets whiles 5 (8.1%) were on anticoagulants, Table 3. All participants on antiplatelets were taking aspirin except one who was on clopidrogrel. Warfarin was the only anticoagulant participants took. Twenty-three (37.1%) of the participants had CSDH on the right, twenty-four (38.7%) on the left CSDH, fifteen (24.2%) had bilateral CSDH Table 3.

**Table 3:** Association between hypertension, diabetes, antiplatelet, anticoagulant, alcohol abuse, laterality and recurrence of CSDH

Condition	Outcome		χ2-value	OR (95% CI)	*P*-value
	No recurrence n (%)	Recurrence n (%)			
Hypertension:			4.251	4.9(0.97–24.28)	0.039
No	23 (92.0)	2 (8.0)			
Yes	26 (70.8)	11 (29.7)			
Total	49 (79.0)	13 (21.0)			
Diabetes:			0.090	1.3(0.23–7.36)	0.670
No	43 (79.6)	11 (20.4)			
Yes	6 (75.0)	2 (25.0)			
Total	49 (79.0)	13 (21.0)			
Antiplatelet:			1.813	2.60(0.62–10.82)	0.226
No	41 (82.0)	9 (18.0)			
Yes	7 (63.6)	4 (36.4)			
Total	48 (79.0)	13 (21.0)			
Anticoagulant:			0.003	0.94(0.09–9.18)	1.000
No	45 (78.9)	12 (21.1)			
Yes	4 (80.4)	1 (20.0)			
Total	49 (79.0)	13 (21.0)			
Alcohol abuse:			0.613	2.05(0.33–12.64)	0.597
No	45 (80.4)	11 (19.6)			
Yes	4 (66.7)	2 (33.3)			
Total	49 (79.0)	13 (21.0)			
Laterality:			7.886	5.98(1.59–22.56)	0.005
Bilateral	8 (53.3)	7 (46.7)			
Left	19 (79.2)	5 (20.8)			
Right	22 (95.7)	1 (4.3)			
Total	49 (79.0)	13 (21.0)			

**Fig. 1:**
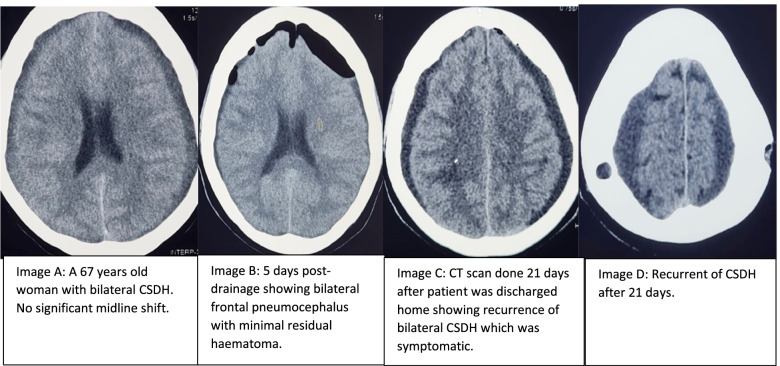
CT scan images of a 67 years old woman with bilateral CSDH before and after burr-hole and drainage surgery

The mean Glasgow Coma Scale (GCS) of the participants was 14 ± 2.5SD. The worst recorded GCS on admission was 5 and the best was 15. The mean INR (international normalized ratio) of the participants before surgery was 1.1 ± 0.2SD. The lowest recorded INR was 0.9 and the highest was 1.8 with a standard deviation of 0.2. The mean platelet count of the participants prior to surgery was 254.3 ± 78.2SD. The lowest recorded platelet count was 119 and the highest 496. The mean size of the haematoma was 2.1 ± 0.6SD cm. The smallest size of the haematoma was 1.0 cm and the largest was 3.0 cm. The largest haematoma was recorded on the right in a participant with bilateral lesion. The mean midline shift was 0.6 ± 0.3SD cm. Some lesions did not cause a midline shift. The reported midline-shift of zero was mostly observed in bilateral lesions as compared to the unilateral ones. The highest reported midline shift was 1.5 cm. Figure 1, shows CT scan images of a 67 years old woman with bilateral CSDH before and after burr-hole and drainage surgery.

The mean duration of admission was 5 ± 2.5 days. The shortest duration of admission was a day whilst the longest was 15 days. Thirteen out of the 62 participants (21.0%) had recurrence of CSDH after the initial evacuation. The median period of recurrence of CSDH was 32.5 days Range 10–90 days).

There was no statistically significant association between age and recurrence (*p* = 0.208). Ten out of the 13 participants (76.9%) who suffered recurrence were males but sex was not statistically significantly associated with recurrence (*p* = 0.495), Table 1.

Twelve participants (19.4%) who presented with facial palsy had recurrence of chronic subdural haematoma. The association between a presentation with facial palsy and recurrence of CSDH was statistically significant (*p* = 0.045) Table 2.

Eleven participants (17.7%) who presented with a history of dysphasia had recurrence of CSDH. The association between clinical presentation of dysphasia and recurrence of CSDH was statistically significant (X^2^ = 4.762, *p* = 0.029). The association between a presentation with headache, seizure, hemiparesis and recurrence of CSDH were not significant statistically (*p*-values > 0.05). A high proportion (61.5%) of the participants who were hypertensive had recurrence of CSDH. The association between hypertension and recurrence of CSDH was statistically significant (*p* **=** 0.039, OR = 4.9, CI = 0.9–24.3). The proportion of those who had recurrence with diabetes was 55.7%. The association between diabetes mellitus and recurrence of CSDH was not statistically significant (*p* = 0.670, OR = 1.3, CI = 0.2–7.4). Four (36.4%) of the 11 participants with history of taking antiplatelets had recurrence of CSDH.

The proportion of recurrence amongst those taking antiplatelets was higher compared to those who were not (67.8% versus 44.8%). Antiplatelets intake was not associated with recurrence (*p* = 0.226, OR = 2.6, CI = 0.6–10.8). Within the participants who had recurrence, the proportion of those who took anticoagulant was similar to those who did not (48.1% versus 50.1%). A history of taking anticoagulants was not associated with recurrence of CSDH [*P* = 1.000, OR = 0.9, CI = 0.1–9.2]. Two (33.3%) of the patients with a history of alcohol abuse had recurrence of CSDH. There was a high proportion of recurrence of CSDH amongst those who used alcohol compared to those who did not (66.3% versus 47.9%). However, alcohol use was not significantly associated with recurrence of CSDH [*P* = 0.613, OR = 2.0, CI = 0.3–12.6]. Seven (46.7%) out of the 15 participants who presented with CSDH had recurrence. The proportion of recurrence of CSDH was higher in those with bilateral lesion when compared to those without (76.7% versus 35.6%). Laterality (bilateral CSDH) was significantly associated with recurrence of CSDH (*P* = 0.005, OR = 5.9, CI = 1.6–22.6) Table 3.

After adjusting for the effect of age, hypertension, diabetes, alcohol abuse, antiplatelets ingestion and anticoagulant ingestion and laterality on recurrence of CSDH in a multivariate logistic regression analysis, laterality was the only predictor of recurrence of CSDH [*P* = 0.030, AOR = 5.5 CI =1.2–25.3]. Respondents with bilateral presentations were 5 times more likely to have recurrence than those who had lateral presentation [*P* = 0.030, AOR = 5.5, CI =1.2–25.3] Table 4.

**Table 4:** Predictors of recurrence of CSDH

Risk factors	AOR	95% Confidence Interval	*P*-value
Age	1.00	0.93–1.10	0.842
Sex:			
Female	0.96	0.13–7.17	0.972
Male	Reference category		
Headache	0.55	0.03–8.83	0.674
Seizure	0.30	0.02–4.55	0.386
Hemiparesis	0.91	0.06–13.89	0.947
Facial palsy	0.53	0.01–21.58	0.737
Dysphasia	0.06	0.01–2.73	0.151
Hypertension	0.08	0.01–1.45	0.88
Diabetes Mellitus	0.76	0.14–4.34	0.761
Alcohol abuse	1.40	0.05–45.03	0.849
Antiplatelets ingestion	0.67	0.05–9.60	0.764
Anticoagulant ingestion	1.09	0.11–10.76	0.941
Laterality:			
Bilateral	5.47	1.18–25.34	0.030
Lateral	Reference category		

Assessing the functional status at 3 months using the modified Rankin score, 52 participants (83.9%) recovered fully after surgery. Six (9.7%) had some symptoms but were not disabled. About 94 % (93.6%) of the patients had good functional outcome at 3 months. One (1.6%) participant had moderate disability. The mortality was 4.8%.

Using the Glasgow outcome scale, fifty-eight (93.6%) patients had a score of 5 which represents full recovery. One (1.6%) participant had a score of 4. Therefore 95.2% of the patient had good recovery 3 months after surgery. Three deaths (4.8%) were recorded.

*AOR* Adjusted Odds Ratio.

## Discussion

Chronic subdural haematoma is a common neurosurgical emergency. An improved life expectancy in a population is associated with an increase in the incidence of chronic subdural haematoma [4]. This study was conducted to determine the factors that predicts the recurrence of CSDH. A total of 62 participants were studied within a year which was relatively higher than the 96 participants that were studied in 4 years, at the same study site 20 years ago [15]. This relative increase in the cases of CSDH may reflect the improved life expectancy of Ghanaians which has resulted in an increase in the number of elderly citizens [18]. Improvement in the availability of diagnostic equipment such as CT scan and MRI could be attributed to this increase.

There was a male preponderance of 72.6% over females of 27.4% with a male to female ratio of 2.6:1. This preponderance of males in CSDH may be attributed to the greater exposure of males to injury [19]. A study has attributed the lower incidence of CSDH in females to the protective effect of estrogen on capillaries and the relatively wider intracranial radius in males [19]. The male to female ratio was similar to those reported in other studies in West Africa and other parts of the world [4, 16, 20]. It was however different from the findings of Dakurah et al [17], in a retrospective study done 20 years ago at the same study site where a male to female ratio of 16:1 was reported. This may be due to a relatively high number of injuries from road traffic accidents (RTA) in their studies. RTA from motorbikes and motorcycles are common amongst young males in West Africa.

Majority (59.7%) of the participants were hypertensives. Hypertension was found to be a major risk factor in other studies from west Africa. However, the percentage of hypertensives in this study is much higher than previous studies done in west Africa. Dakurah et al had 9.7% in Accra, Bankole et al had 22.9% in Lagos, Jimoh et al had 23.3% in Zaria and Chikani et al had 15.87% in Enugu [16, 17, 20–23]. The higher percentage of hypertensives found in this study is however consistent with a recent multi-centre report from Eastern Europe which indicated that as high as 75% of patients with CSDH were hypertensives [24]. It has been suggested that hypertension predisposes to spontaneous intracranial bleeds and this could be responsible for its high prevalence in patients with CSDH. This prevalence increases with age. The use of antithrombotic agents amongst the participants is similar to other studies. However, Rust *et al* reported that up to 23% of their participants were on antithrombotic agents [25]. Eight (12.9%) of the participants were diabetics. Five out of the 8 diabetics also had a history of hypertension. The frequency of participants with a history of diabetes in this study is one of the highest in West Africa [16]. The prevalence of diabetes mellitus amongst patients reporting with CSDH is less than 10%. Dakurah et al. had no diabetics in their series whilst Chikani and Ozor reported a prevalence of 3.4% [17, 23]. These figures do not reflect the increase tendency of falls within diabetics as reported by Wang et al. [26] They reported that diabetics had about 2-fold increase in developing CSDH compared to non-diabetes cohort (2.04 vs. 1.30 per 1000 person-years), with an adjusted hazard ratio of 1.63 [95% CI (1.43–1.85].

Six (9.7%) of the participants had a history of alcohol abuse. This is similar to the findings in the series by Dakurah et al. but lower than other studies in Africa [17]. Bankole et al series indicated a significant alcohol use in 43.3% of the participants [20]. Kitya et al reported a prevalence of alcohol abuse in 26.8% of the participants in Mbarara, Uganda [27].

Regarding the laterality of the haematoma, bilateral lesions accounted for 24.2%. This is similar to other studies but differ from the findings of Dakurah et al of 11.5% [17]. The unilateral lesions were made up of 38.7% on the left and 37.1% on the right. All the participants who presented with bilateral CSDH were above the mean age of 63.1 years. Bilateral CSDH tend to occur in the elderly and it has been suggested that this may be due to cerebral atrophy [19].

The recurrence rate of CSDH for this study was 21.0%. This is very high when compared to the recurrence rate of 2.1% in the series by Dakurah et al. [17] The difference between the mean age of the two studies (63.1 versus 46.9 years) was statistically significant (one sample test value 9.392, *P* < 0.001, CI = 19.606). High recurrence rate have been observed amongst the elderly. This has been attributed to cerebral atrophy which exposes the elderly to falls and CSDH. Regarding other studies in West Africa, the recurrence rate in this series is still high [16]. It is however, comparable to other studies from Europe and Asia where there is generally a higher population of elderly people [28–30]. This might also reflect the improvement in the availability of diagnostic equipment such as CT scan and MRI which enhances the evaluation of recurrence. Ten out of the 13 participants who had recurrence were males. However, the association between the gender of the participants and recurrence was not statistically significant (*P* = 0.495). This might be due to the high frequency of males in this study. The participants who were 60 years and above accounted for as high as 87.6% of recurrence. Recurrence was not observed in those below 50 years. This is consistent with the high prevalence of recurrence amongst the elderly reported in other studies [30]. It was also observed that as a high proportion (60.9%) of those who presented with facial palsy developed recurrence. The association between a presentation with facial palsy and recurrence of CSDH was statistically significant (X^2^ = 5.033, *P* = 0.045). Similarly, there was a high proportion (62.38%) of recurrence within the participants who presented with dysphasia. This association was statistically significant. This finding is similar to a study by Hammer et al. which indicated that the association of aphasia with recurrence of CSDH was statistically significant. (*P* = 0.008; Χ^2^ – independent T-test) [31]. However, no reason was adduced for the findings by Hammer and his colleagues. Though greater than 80% of the participants had a history of headache, its association with recurrence of CSDH was not statistically significant. There was no statistically significant association between a presentation with seizure nor hemiparesis and recurrence. These findings are however contrary to the series of Kim et al. which found a statistically significant association between headache and recurrence of CSDH [32]. Jung et al. also reported a statistically significant association between hemiparesis and recurrence of CSDH (*P* = 0.026) [33]. The risk of a hypertensive developing recurrence of CSDH was about 5-times of a non-hypertensive. This is consistent with numerous studies which found hypertension as risk factor for the development of recurrence after an initial surgery for CSDH [34, 35]. All but one participants with hypertension who developed recurrence was over 70 years. Considering the fact that higher recurrence was observed in the participants who were over 70 years, it may imply that age was a confounding factor. Therefore, following a multivariate analysis using logistic regression, hypertension did not show a statistically significant association with recurrence of CSDH. This explains the fact that the observation in the univariate analysis was confounded by the high prevalence of participants who were over 70 years. From the study, diabetics were about 3 times (2.6) more likely to have recurrence of chronic subdural haematoma than non-diabetics. However, from a multivariate logistic regression analysis, diabetes was not an independent risk factor for the development of recurrent CSDH. This is consistent with several studies that did not found any statistically significant association between diabetes mellitus and recurrence of CSDH [13, 36]. It was suggested by Yamamoto et al. that diabetes actually decreases the risk of recurrence of CSDH [36]. They postulated that diabetes increases the viscosity of blood and platelet aggregation and eventually enhances coagulation which decreases the risk of re-bleeding [36].

This study did not show any statistically significant association between alcohol abuse and recurrence of CSDH. Alcohol abuse is a known risk factor for cerebral atrophy which is a predisposing factor for CSDH. Alcohol use also impairs platelet function and thus reduces coagulation. Alcohol users are at risk of multiple falls which further predisposes them to spontaneous intracranial bleed. It is thus expected that alcohol use will increase the risk of recurrent CSDH. The results of this study is at variance with that of Schmidt et al., who reported a statistically significant association between alcohol use and recurrence [37]. It is however similar to other studies which did not find statistically significant association between alcohol abuse and recurrence of CSDH [38, 39].

Both the pre-operative platelet counts and INR of the participants who were on antiplatelet therapy were within the normal range. None of these participants required platelet transfusion. All of them stopped antiplatelet therapy prior to surgery and recommenced after a month during the post-operative period. This study did not show any statistically significant association between the antiplatelet therapy and recurrence of CSDH. Following a multivariate logistic regression analysis, antiplatelet therapy was not seen as a predictor of recurrence of CSDH. This is consistent with previous studies which did not find antiplatelet therapy as a predictor of recurrence [11, 40, 41]. In a recent UK-based multi-centre prospective cohort study involving 817 participants antiplatelet therapy was not found to be associated with recurrence of CSDH [11]. It has been further recommended that antiplatelet therapy should be resumed within a week after surgical intervention in patients with high risk of cerebrovascular or cardiovascular thrombotic effect [11]. The findings of this current study further enhances this recommendation.

There was no statistically significant association between anticoagulant therapy and recurrence of CSDH. This finding are consistent with recent studies which indicated that anticoagulant therapy was not associated with recurrence of CSDH [11]. It was observed further that cessation of anticoagulant therapy was associated with thrombotic events [11].

The prevalence of recurrence among the participants who had bilateral lesions was very high (76.7%) compared to those who had unilateral lesions. Bilateral CSDH had a statistically significant association with recurrenc﻿e. Participants with bilateral CSDH were 6 times more likely to develop recurrence. Thus bilateral CSDH proved to be the only independent predictor of recurrence. Bilateral CSDH has been found to be prevalent in patients with cerebral atrophy [42]. Cerebral atrophy results in the stretching of the bridging veins which thins the walls and predisposes these individuals to CSDH. It has therefore been found to be associated with recurrence of CSDH and the findings of this study is consistent with other numerous studies which reported same [13, 39, 43, 44].

Assessing the functional outcome of the participants at 3 months, 93.6% of them had a good outcome. The mortality rate for this study was 4.8%. All the 3 mortalities were recorded in patients who were over 60 years. Two of them were above 70 years. These 2 participants presented with a GCS of 7 and 9. One eventually died from aspiration Pneumonia after an initially improvement in the GCS. The other patient who was an 83-year-old diabetic developed recurrence and later complicated by subdural empyema. The multiple surgeries and co-morbidity of diabetes might have predisposed him to the deep surgical site infection which eventually led to his demise. The mortality rate for this study is below the reported average of 11.0%. This is however higher than a rate of 2.1% recorded by Dakurah et al. [17] In a recent prospective studies conducted in Mbarara, Uganda, a mortality rate of 5.6% was recorded [27]. The peri-operative mortality rate for CSDH in West Africa is between 2.0% to 12.5% with the highest recorded in Lagos by Bankole et al [20]. Although the outcome of this study supports the institution of measures to reduce the burden of CSDH, and improve the quality of life of patients, few limitations such as the relatively small number of participants on antiplatelets and anticoagulants and the period of assessing recurrence (3 months) may exclude those who may develop recurrence after 90 days.

## Conclusion

The only independent predictor of recurrence of chronic subdural haematoma was bilaterality of the lesion. Both hypertension and bilaterality had a statistically significant association with recurrence of chronic subdural haematoma. Patients with bilateral chronic subdural haematoma must be followed closely after treatment with frequent sensitive test (CT scan) to pick recurrent disease before it becomes fatal. The resumption of anti-thrombotic therapy must be instituted as early as possible for highly risks patients such as those with past history of myocardial infarction, pulmonary embolism, DVT and cerebrovascular accidents since it appears taking these agents does not increase the re-operation rate.

## Supplementary Information

**Additional file 1.**

## Data Availability

The datasets used and/or analysed during the current study are available from the corresponding author on reasonable request. The raw data set have been included as a supplementary document in the submission page.

## Abbreviations

AOR: Adjusted Odds Ratio
CSDH: Chronic Subdural Haematoma
CSF: Cerebrospinal fluid
CT: Computerised Tomography
GCS: Glasgow Coma Score
GOS: Glasgow outcome Score
IBM: International Business Machine
INR: International Normalised Ratio
KBTH: Korle-bu Teaching Hospital
MRI: Magnetic Resonance Imaging
MRS: Modified Rankin scale
RTA: Road traffic accident
SEP: Subdural Evacuation Port
SPSS: Statistical Package for the Social Sciences
VEGF: Vascular Endothelial Growth Factor
VP: ventriculoperitoneal
